# Orthologs of Human Disease Associated Genes and RNAi Analysis of Silencing Insulin Receptor Gene in *Bombyx mori*

**DOI:** 10.3390/ijms151018102

**Published:** 2014-10-09

**Authors:** Zan Zhang, Xiaolu Teng, Maohua Chen, Fei Li

**Affiliations:** 1 Department of Entomology, College of Plant Protection, Nanjing Agricultural University, Nanjing 210095, China; E-Mails: zhangzan125@gmail.com (Z.Z.); tengxiaolu177546@gmail.com (X.T.); 2 College of Plant Protection, Northwest A&F University, Taicheng Road, Yangling 712100, Shaanxi, China; E-Mail: cmhwh1@hotmail.com

**Keywords:** *Bombyx mori*, human diseases model, diabetes mellitus, INSR, dsRNA

## Abstract

The silkworm, *Bombyx mori* L., is an important economic insect that has been domesticated for thousands of years to produce silk. It is our great interest to investigate the possibility of developing the *B. mori* as human disease model. We searched the orthologs of human disease associated genes in the *B. mori* by bi-directional best hits of BLAST and confirmed by searching the OrthoDB. In total, 5006 genes corresponding to 1612 kinds of human diseases had orthologs in the *B. mori*, among which, there are 25 genes associated with diabetes mellitus. Of these, we selected the insulin receptor gene of the *B. mori* (*Bm-INSR*) to study its expression in different tissues and at different developmental stages and tissues. Quantitative PCR showed that *Bm-INSR* was highly expressed in the Malpighian tubules but expressed at low levels in the testis. It was highly expressed in the 3rd and 4th instar larvae, and adult. We knocked down *Bm-INSR* expression using RNA interference. The abundance of *Bm-INSR* transcripts were dramatically reduced to ~4% of the control level at 6 days after dsRNA injection and the RNAi-treated *B. mori* individuals showed apparent growth inhibition and malformation such as abnormal body color in black, which is the typical symptom of diabetic patients. Our results demonstrate that *B. mori* has potential use as an animal model for diabetic mellitus research.

## 1. Introduction

Animal models are a helpful for studying molecular mechanisms of human disease as well as for drug testing. The most widely used animal models are mouse, *Mus musculus* and Rat, *Rattus rattus*, which have been used to model many human diseases, including neurological and behavior disorders [1,2,3,4]. The advantages of using rodents as disease models are apparent. These mammals are evolutionally close to humans and share similar gene regulation mechanisms. Transgenic mice have become an efficient method to produce a disease model [5]. Besides mouse, the dog was used to model human non-Hodgkins lymphoma [6]. However, mouse, rat and dog have a long life cycle and the costs are high. To overcome these disadvantages, the fruitfly, *Drosophila melanogaster*, was widely used for modeling lots of human disorders such as neurodegenerative diseases [7,8,9], Huntington’s disease [10], Barth syndrome [10,11], Parkinson’s disease [12], Alzheimer’s disease [13], cancer [14], heart disease [15,16,17], and Multiple endocrine neoplasia [18]. The success of using insect models for human diseases is exciting and highly useful since it provides a cost-effective strategy in disease research and drug screening.

However, it is impossible to use one animal to model all kinds of human disorders and choosing the right animal model is important. The choice often considers the nature of the disease and characteristics of the animal [19,20,21]. The *B. mori* genome was published in 2004 and many *B. mori* genes have been well studied [22,23]. The advantages of using the silkworm to model human diseases are apparent: (1) The life cycle of silkworm is about one month, which is much shorter than mice and rats. The shorter life cycle will accelerate the research; (2) Since the *B. mori* has been domesticated for more than two thousand years, it is very easy to be maintained in the lab with artificial diets; and (3) Transgenic *B. mori* is easy to be obtained with routine methods [24,25,26]. The silkworm has been widely used as the bioreactor to express human genes for producing drugs or vaccines [27,28,29]. Because of these advantages, the *B. mori* has been successfully used to model human disorders such as Parkinson’s disease [30], and human sepiapterin reductase deficiency [31]. The *B. mori* was also helpful in screening drugs. It was successfully used to screen ant-diabetic drugs by feeding a high glucose-containing diet [32]. Five proteins were induced by a high-glucose diet in the silkworm [33]. Bombyxin is an insect insulin-related peptide. Bombyxin can reduce the concentration of the major hemolymph sugar trehalose in larvae [34,35]. Glucose can stimulates the release of Bombyxin in *B. mori* [36]. These studies suggest that it is possible to use the *B. mori* to model insulin-related human diseases such as diabetes. Here, we searched the orthologs of human disease-associated genes in the *B. mori*, and then selected the insulin receptor gene in *B. mori* (*Bm-INSR*) for further gene expression and RNAi analysis.

## 2. Results and Discussion

### 2.1. Orthologs of Diseased-Associated Genes in the B. mori

**Figure 1:**
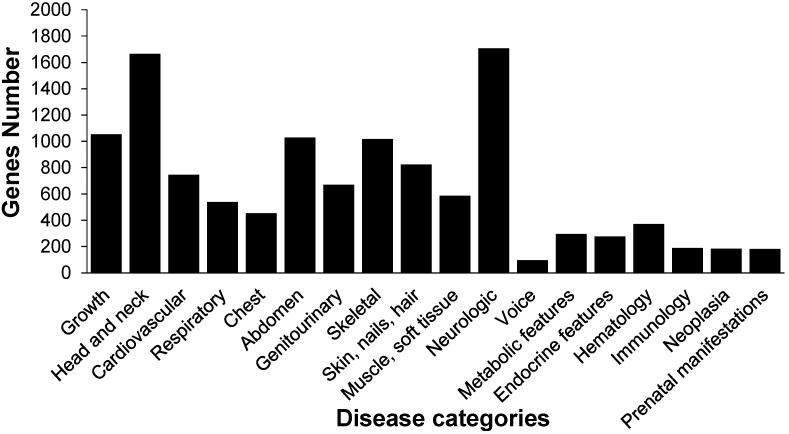
The numbers of the silkworm orthologs in different categories of human diseases.

We obtained 69,040 protein sequences corresponding to 2629 kinds of human disorders from the database of Online Mendelian Inheritance in Man (OMIM) [37]. The 14,623 *B. mori* genes were downloaded from the SilkDB (v2.0). These genes contain intact open reading frames (ORF). We identified orthologs by using bidirectional best hits, which is a widely used method. The *E*-value cutoff of Blast is 0.00001. As a result, 5006 *B. mori* genes were orthologs of human disease-associated genes, corresponding to 1612 kinds of human disorders. We classified human diseases into 18 categories, showing that silkworm (*B. mori*) orthologs of human disease-associated (SOHD) genes are mainly related with skeletal disease, head and neck disease, neurologic disease, and growth diseases (Figure 1, Table S1) [38]. In these SOHD genes, 4941 *B. mori* genes have orthologs in *D. melanogaster*, supporting the utility of *B. mori* as an alternative model (Table S1).

**Figure 2:**
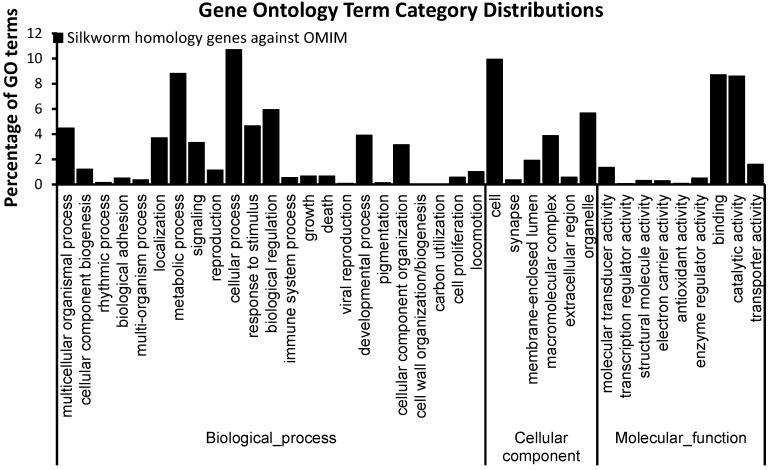
Gene ontology (GO) term classifications of silkworm orthologs of human disease. GO analysis was performed using Blast2go. The GO terms were classified into different categories at level 2.

**Figure 3:**
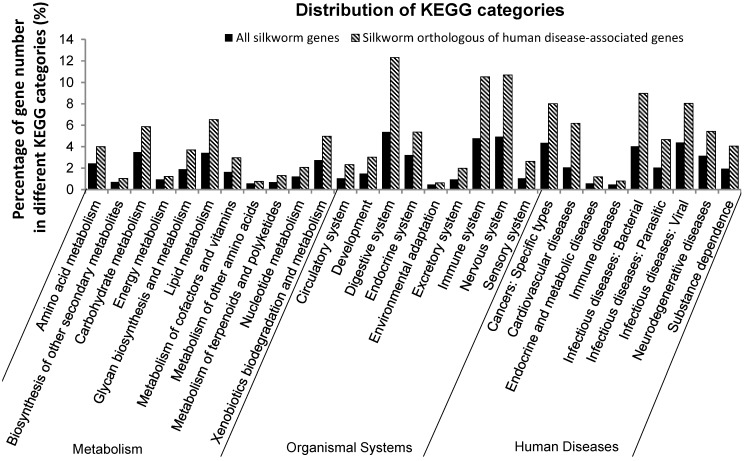
Pathway analysis of silkworm genes associated with human disease. The analysis was performed using BLASTP against KEGG database. The pathways were clustered with three different systems, metabolism system, organismal system and human disease.

Gene ontology analysis showed that SOHD genes were rich in “symplast” at the cellular component system but lacked “protein tag” and “nutrient reservoir activity” at the molecular function system (Figure 2). Pathway analysis indicated that SOHD genes were rich in carbohydrate metabolism and lipid metabolism. For organismal system, SOHD genes were rich in digestive system, immune system and nervous system. For human disease system, SOHD genes were rich in infectious, cardiovascular and cancer (Figure 3).

### 2.2. Insulin Receptor Genes in the B. mori

We found that 25 *B. mori* genes were orthologs of human genes associated with diabetes mellitus (Table 1). Insulin receptor-like protein precursor in the *B. mori* (*Bm-INSR*, SilkDB ID: BGIBMGA003654) shares 56% sequence similarity with human insulin (GenBank ID: EAW69046). Domain analysis showed that *INSR* genes in human, mouse and *B. mori* were highly conserved. They all contained two receptor L domains (Receptor L), furin-like repeats (cysteine-rich region), fibronectin type 3 domain (FN3) and protein kinases catalytic domain (PTKc) [39,40], indicated that they had the similar function. Thus, we selected the *Bm-INSR* for further analysis.

**Table 1:** The silkworm orthologs of human genes associated with diabetes mellitus.

Accession Numbers of Human Genes	SilkDB ID	*E*-Value	Diabetes Disorders
NP_005318.3	BGIBMGA007360	2.00 × 10^−89^	Hyperinsulinemic hypoglycemia, familial, 4, 609975.
NP_619726.2	BGIBMGA006839	5.00 × 10^−28^	Insulin resistance, severe, digenic, 604367. Obesity, resistance to Diabetes, type 2, 125853.
EAW69046.1	BGIBMGA003654	0	Diabetes mellitus, insulin-resistant, with acanthosis nigricans, 610549. Hyperinsulinemic hypoglycemia, familial, 5, 609968.
NP_001073285	BGIBMGA003800	2.00 × 10^−64^	
BAC11220.1	BGIBMGA009492	4.00 × 10^−22^	Diabetes mellitus, noninsulin-dependent 1, 601283.
NP_000343.2	BGIBMGA006882	9.00 × 10^−50^	Diabetes mellitus, noninsulin-dependent, 125853. Diabetes mellitus, permanent neonatal, 606176. Diabetes mellitus, transient neonatal 2, 610374. Hyperinsulinemic hypoglycemia, familial, 1, 256450.
NP_005996.2	BGIBMGA002273	1.00 × 10^−35^	Diabetes mellitus, noninsulin-dependent, susceptibility to, 125853.
BAA91102.1	BGIBMGA007364	1.00 × 10^−25^	
NP_001007226	BGIBMGA004315	1.00 × 10^−8^	
NP_006539.3	BGIBMGA007473	1.00 × 10^−7^	
NP_060244.2	BGIBMGA011966	0	
NP_776250.2	BGIBMGA005779	1.00 × 10^−72^	
NP_002818.1	BGIBMGA002096	1.00 × 10^−66^	
AAA52569.1	BGIBMGA010881	1.00 × 10^−8^	
NP_001033.1	BGIBMGA002023	3.00 × 10^−93^	
NP_003042.3	BGIBMGA003138	6.00 × 10^−40^	Hyperinsulinemic hypoglycemia, familial, 7, 610021.
BAF83535.1	BGIBMGA003740	6.00 × 10^−44^	Diabetes mellitus, noninsulin-dependent.
BAH12783.1	BGIBMGA004629	3.00 × 10^−12^	
NP_000331.1	BGIBMGA010740	3.00 × 10^−51^	
NP_005262.1	BGIBMGA014352	1.00 × 10^−38^	Hyperinsulinism-hyperammonemia syndrome, 606762.
NP_002702.2	BGIBMGA011660	3.00 × 10^−19^	Insulin resistance, severe, digenic, 604367.
EAX02222.1	BGIBMGA008597	3.00 × 10^−22^	Insulin-like growth factor I, resistance to, 270450.
NP_000866.1	BGIBMGA008240	3.00 × 10^−19^	
AAA93480.1	BGIBMGA003134	4.00 × 10^−10^	Maturity-onset diabetes of the young 6, 606394. Diabetes mellitus, noninsulin-dependent, 125853.
NP_002491.2	BGIBMGA008390	2.00 × 10^−9^	

### 2.3. The Expression of Insulin Receptor Gene in the B. mori

**Figure 4:**
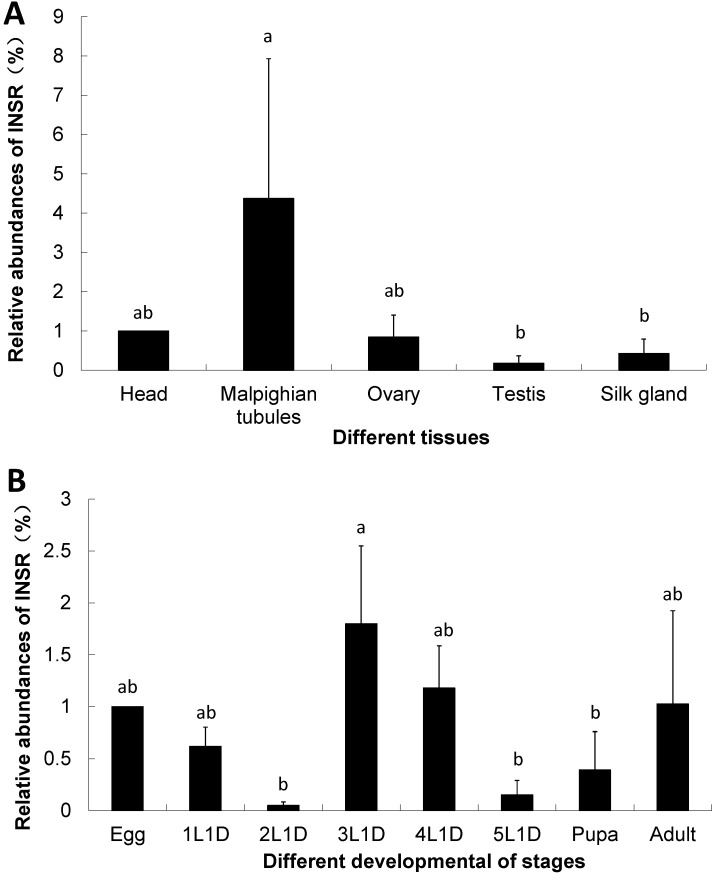
Relative abundance of *Bm-INSR* gene in different tissues and developmental stages. (**A**) different tissues; and (**B**) different developmental stages. 1L1D, 2L1D, 3L1D, 4L1D, 5L1D: First day of each instar (Tukey test, *p* < 0.05. “a” and “b”, same letters in figure differentiated no significantly, different letters differentiated significantly). The gene expression levels were examined using quantitative real time PCR. The gene *RP49* was used as the internal control.

We investigated the expressions of *Bm-INSR* gene in six tissues including head, Malpighian tubules, ovary, testis and silk gland. The housekeeping gene *RP49* was used as the reference gene in qPCR. *Bm-INSR* was highly expressed in the Malpighian tubule but expressed at low levels in the testis (Figure 4A). The Malpighian tubule is a type of excretory and osmoregulatory system. Though it does not have a digestive function, the Malpighian tubule can release the waste of digested food. It has been reported that an H^+^-dependent trehalose transporter can induce the reabsorption of trehalose in Malpighian tubules [41]. This can partially explains the reason of high expression of *Bm-INSR* gene in the Malpighian tubule.

The egg, larvae at the first day of each instar (1L1d, 2L1d, 3L1d, 4L1d 5L1d), pupa and adult *B. mori* were also chosen for analysis. The results showed that *Bm-INSR* gene was highly expressed in the 3rd and the 4th instar larvae, followed by egg and adult. It was expressed at low levels in the 2nd and the 5th instar larvae (Figure 4B). The expression peaks of insulin receptor gene in the 3rd and 4th instars indicated that insulin receptor gene is related with food metabolism because the *B. mori* larvae eats lots of mulberry leaves at these two stages. It has been reported that glucose in the food stimulates the release of Bombyxin in *B. mori* [36].

The expression of the *Bm-INSR* was higher in the Malpighian tubules than in testis and silk gland (Figure 4A). Since the Malpighian tubule is a type of excretory and osmoregulatory system, which can release the food waste, the reabsorption of trehalose was reported in Malpighian tubules. The high expression of *Bm-INSR* gene in this tissue indicated that the *Bm-INSR* is associated with food metabolism.

### 2.4. Knockdown of the Insulin Receptor Genes in the B. mori

**Figure 5:**
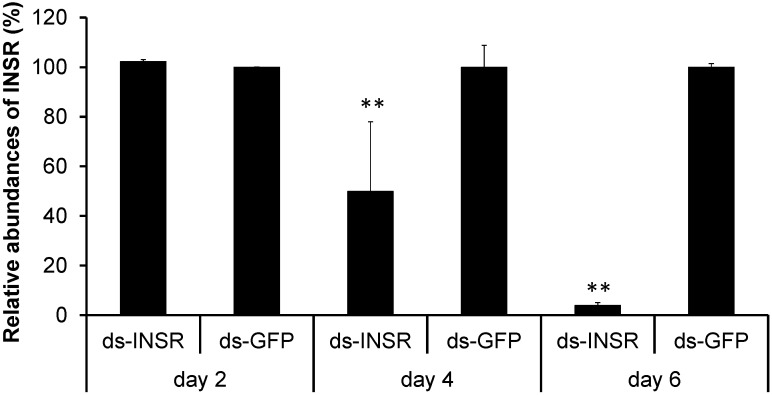
Relative abundance of *Bm-INSR* genes after dsRNA injection. Ds-GFP signifies the negative control groups. ds-INSR represents dsRNA treated groups. Asterisks indicate statistically significant difference (*t*-test, *p* < 0.01). The gene expressions were examined using quantitative real time PCR. The gene *RP49* was used as the internal control.

DsRNA was designed based on *Bm-INSR* gene sequence and then was synthesized using T7 RiboMAX kit (Promega, Madison, WI, USA). One μL of dsRNA (5000 ng·μL^−1^) was injected into the 3rd instar larvae. The dsRNA designed from *GFP* gene sequence was used as the negative control. To examine RNAi efficiency, we collected RNAi-treated larvae at 48, 96, and 124 h after dsRNA-injection. Real time PCR analysis showed that the abundance of *Bm-INSR* gene was decreased to 50.2% of the control level at 96 h post-injection (Figure 5). The mRNA abundance of the *Bm-INSR* gene dramatically decreased to 4% at 124 h post-injection (*p* < 0.05). The results showed that we silenced the *Bm-INSR* successfully.

### 2.5. The Impact of INSR Gene Knockdown on B. mori Development

**Figure 6:**
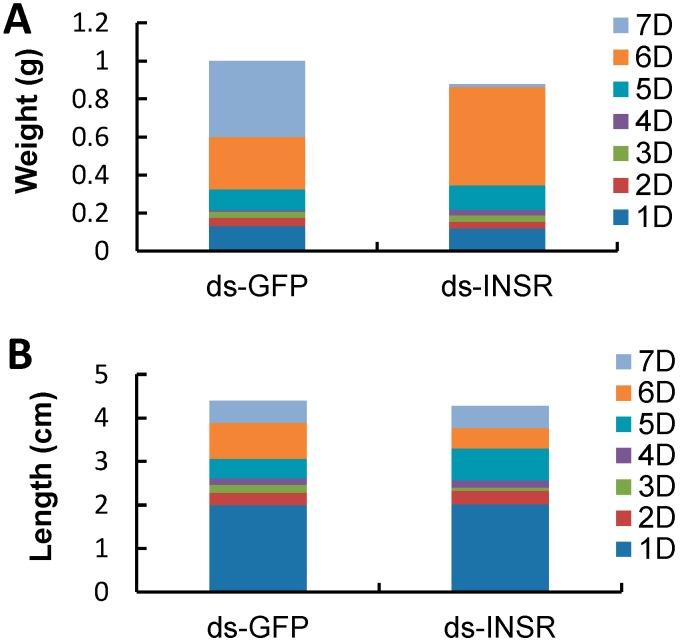
The change of the body length and weight in larvae of different treatments after injection. Ten animals were used in each experiment. Ds-GFP: Group after injection the dsRNA used to silence *GFP*. ds-INSR: Group after injection the dsRNA used to silence INSR. 1D, 2D, 3D, 4D, 5D, 6D, 7D: One days, two days ,three days ,four days, five days, six days and seven days after injection. (**A**) shows the change of weight of the insect body in 7 days and (**B**) shows the change of length of the insect body in 7 days.

**Figure 7:**
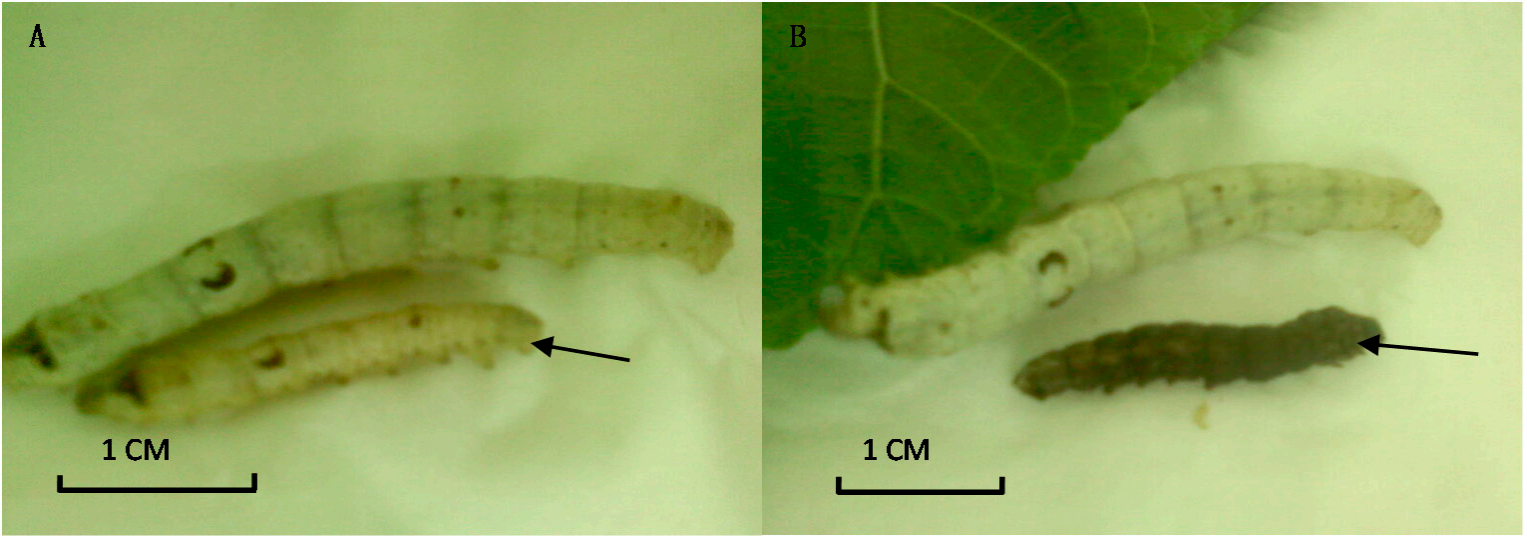
Growth inhibition of dsRNA-treated larvae. (**A**) The growth retardation compared with the control at 10 days after injection; and (**B**) The RNAi-treated silkworm in black body color at 10 days after injection.

We chose ten RNAi-treated *B. mori* individuals to measure their weights and lengths. At seven days after dsRNA injection, the average body weight of RNAi-treated groups is 0.879 ± 0.021 g, which was less than that of the control group (1.00 ± 0.35 g). The significant difference was observed between the third and sixth days post-injection (*p* < 0.01, *t*-test, Figure 6A). However, knockdown of the *Bm-INSR* gene did not affect body length. At seven days post-injection, the average length of the RNAi-treated group was 4.27 ± 0.57 cm, similar to the control group (4.4 ± 0.46 cm) (Figure 6B). In the RNAi-treated group, 20% of individuals showed growth retardation. About 20% of RNAi-treated *B. mori* became black in body color (Figure 7). The mRNA levels of *Bm-INSR* gene were significantly decreased in the silkworm that became black in body color, which might be associated with melanin synthesis, because there was not any silkworm that became black in the control group. So, it is unlikely that RNAi induces the body color change. It has been reported that abnormal pigmentation is typically a sign of an innate immune response and insulin signaling is closely connected with immunity [41,42,43].

The results indicated that dsRNA-treated silkworms showed two typical symptoms of diabetes patients. One was weight reduction, and the other was black body color. These results demonstrated that *Bm-INSR* has important functions in silkworm development and *B. mori* could be used as an animal model for diabetes mellitus by RNA interference.

## 3. Experimental Section

### 3.1. Insects

The *B. mori* (strain: 1079) were maintained using mulberry leaves under the condition of 80% relative humidity and 25 °C. For gene expression analysis, egg, larvae at the first day of each instar, pupa and adult *B. mori* were collected. The tissues of head, ovary, testis, Malpighian tubules and silk glands were dissected from the 5th instar larvae. All samples were frozen using liquid nitrogen and then kept at −70 °C for RNA purification. All experiments were done in triplicates.

### 3.2. RNA Purification and cDNA Synthesis

The samples were frozen with liquid nitrogen and homogenized in a tissue grinder. Then, 1 mL TRIzol reagent (Invitrogen, Carlsbad, CA, USA) was added. Total RNA was isolated following the manufacturer’s instructions. DNA was removed from total RNA by DNase I treatment following the protocol (Takara, Otsu, Japan). RNA integrity was checked on a 1.0% agarose gel and visualized by ethidium bromide staining. The concentration and quality of RNA were measured with Nano drop ND-1000 (Thermo scientific, Wilmington, DE, USA). The first strand of cDNA template was synthesized from 1 μg total RNA using M-MLV reverse transcriptase (Takara, Otsu, Japan) and Oligo (dT18) as the anchor primers. The reaction mixture was incubated at 70 °C for 10 min followed by 42 °C for 1 h and 70 °C for 15 min.

### 3.3. Quantitative Real-Time PCR

The quantitative real-time PCR (qPCR) reactions were carried out with SYBR Premix Ex Taq™ (Takara, Otsu, Japan) following the manufacturer’s protocol using an ABI Prism 7300 (Applied Biosystems, Foster City, CA, USA). The reaction mixture contained 2 μL cDNA template. The final reaction volume was 20 μL. The PCR reactions included an initial step of 95 °C for 30 s, followed by 40 cycles of 95 °C for 5 s and then annealed at 60 °C for 31 s, one cycle of 95 °C for 15 s, 60 °C for 60 s and 95 °C for 15 s. The primers were designed with Beacon designer 7.0 (PREMIER Biosoft, Palo Alto, CA, USA) (Table 2). Amplification efficiencies were determined by template dilution. The qPCR specificity was monitored with melting curve analysis using the SDS software (2.2.1, Applied Biosystems, Foster City, CA, USA) and gel electrophoresis. The housekeeping gene *RP49* was used as the reference genes for data normalization. The relative abundance was calculated using 2^−ΔΔ*C*t^ method [44]. The data were analyzed using the SPSS software (v19, SPSS, Chicago, IL, USA) (*p* < 0.05). All experiments were repeated in triplicates.

**Table 2:** The silkworm orthologs of human genes associated with diabetes mellitus.

Genes	Primer Names	Sequences
*Bm-INSR*	INSR-qRT-up	5'-GGTCCGAGATGACTTGTATGG-3'
	INSR-qRT-down	5'-GCTGCTGTTGAATGTCTTATAGG-3'
*Ribosomal protein 49*	rp49-qRT-up	5'-AGGCATCAATCGGATCGCTATG-3'
	rp49-qRT-down	5'-TTGTGAACTAGGACCTTACGGAATC-3'
*Bm-INSR*	INSR-dsRNA-up *	5'- 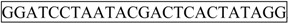 AGACGCTATCATTGTTGGA-3'
	INSR-dsRNA-down	5'- 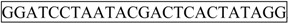 TGCGGTTCACTTTACGA-3'
*GFP*	GFP-dsRNA-up	5'- 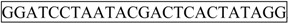 AAGTTCAGCGTGTCCG-3'
	GFP-dsRNA-down	5'- 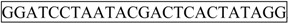 CACCTTGATGCCGTTC-3'

* The sequences in box are the T7 promoter sequence for synthesis of dsRNA.

### 3.4. Double Strand RNA Synthesis

Double strand RNA (dsRNA) was synthesized using T7 RiboMAX kit (Promega, Madison, WI, USA) following the manufacturer’s protocol. The primers for amplifying dsRNA were designed according to the sequence of *Bm-INSR* (BGIBMGA003654) with primer premier 5.0 (Table 2). The PCR products were confirmed by sequencing. The dsRNA of *GFP* genes were used as the negative control.

### 3.5. DsRNA Injection and Phenotype Observation

For RNAi of *Bm-INSR* gene, 1 μL dsRNA (5000 ng·μL^−1^) were injected into the 3rd instar larvae using micro-needles. Each group contained 50 insects. At 24 h after dsRNA injection, ten insects were selected from each group to measure body length and weight. Since then, phenotype observations were carried out every day until pupation. At 48, 96 and 124 h after dsRNA injection, six insects were randomly selected from each group to examine the abundance of *Bm-INSR* gene.

### 3.6. Bioinformatics Analysis

The accession numbers of human disease associated genes were downloaded from the database of Online Mendelian Inheritance in Man (OMIM) [37]. The protein sequences were obtained from the GenBank. The *B. mori* gene sequences were downloaded from the SilkDB database [45]. The orthologs were determined using bidirectional best hits (BBHs) of BLAST [46], which is a widely used method for identifying orthologs. The *E*-value cut-off is 0.00001. The results were confirmed by searching the OrthoDB [47]. The domain analysis of *INSR* genes was conducted by CD-searching. Gene ontology analysis was conducted using the software Blast2go [48]. Pathway analysis was conducted by BLASTP against the KEGG database.

## 4. Conclusions

Animal models have been widely used in studying molecular mechanisms of human diseases and drug screens. Among these animal models, insects have received increasing attention because of short life cycles and convenience of rearing. Many successful experiments have been carried out in *D. melanogaster*. The fruitfly was used to model human diseases such as Alzheimer’s disease [49], Parkinson disease [50], learning and memory [51], and insulin resistance [52,53]. The diamondback moth, *Plutella xylostella*, was used to study excess fat storage under different nutritional environments [54].

However, only several well-known insects were considered for developing disease model. There are many more economical or agricultural important insects with genome sequences available. *B. mori* has been domesticated for thousands of years [55]. The genome of *B. mori* was published almost ten years ago [23]. The silkworm has been used in traditional Chinese medicine for a very long time [56,57]. *B. mori* is also an efficient bioreactor to produce vaccine and drug-related proteins [29,58,59]. In this work, bioinformatics analysis demonstrated that more than 5000 *B. mori* genes were the orthologs of human disease associated. These *B. mori* genes are rich in metabolic pathways, suggesting that *B. mori* might be a suitable animal model for metabolic syndrome and associated complications.

In the ancient medical texts of traditional Chinese medicine, diabetes-related symptoms were called as “Xiaoke” disease. Reference mining of medical texts such as *Huang Di Nei Jing*, the *Yellow Emperor’s Inner Classic*, and approximately 1200 recipes and 150 herbs for diabetes demonstrated that *B. mori* and its moths were widely used to treat “Xiaoke” patients in ancient China [60,61,62,63]. Here, we chose *Bm-INSR* gene as an example to study the possibility of using *B. mori* as diabetes model. Expression analysis showed that *Bm-INSR* was highly expressed at the 3rd and the 4th instar larvae, which was consistent with eating habit of the *B. mori*. RNAi of *Bm-INSR* resulted in high percentages of black body color, which is similar in diabetes patients.

Bombyxin in *B. mori* is the first insulin-like peptide discovered in insects, which shares 56% amino acid similarity with human insulin [64]. The insulin/insulin-like signaling pathways are highly conserved between vertebrate and invertebrate, which is an important basis of using the *B. mori* to model diabetes [65]. The insulin receptor is associated with diabetes in human, however, there is not enough data from insects; this is worthy of further investigation. In summary, we presented experimental evidence that *B. mori* is a suitable animal model for diabetes mellitus. The “diabetes silkworm” can be easily obtained by RNA interference.

## Supplementary Materials

Supplementary materials can be found at http://www.mdpi.com/1422-0067/15/10/18102/s1.

## Supplementary Files

Supplementary File 1
